# Supplementary material for: Passive vs. active warm-up combined with stretching on hamstring flexibility and maximal voluntary contractions
Source: PLoS One. 2025 May 7;20(5):e0322582. doi: 10.1371/journal.pone.0322582 (PMC12057887; doi:10.1371/journal.pone.0322582)
Supplement: Attachment — Submitted filename: responses.docx [file pone.0322582.s003.docx]

**Response to Editorial Team**

Journal Requirements:

When submitting your revision, we need you to address these additional requirements.

1. Please ensure that your manuscript meets PLOS ONE's style requirements, including those for file naming. The PLOS ONE style templates can be found at

https://journals.plos.org/plosone/s/file?id=wjVg/PLOSOne_formatting_sample_main_body.pdf and

<https://journals.plos.org/plosone/s/file?id=ba62/PLOSOne_formatting_sample_title_authors_affiliations.pdf>

**Response:** The Formatting has been verified and modified when necessary

2.  Please include a complete copy of PLOS’ questionnaire on inclusivity in global research in your revised manuscript. Our policy for research in this area aims to improve transparency in the reporting of research performed outside of researchers’ own country or community. The policy applies to researchers who have travelled to a different country to conduct research, research with Indigenous populations or their lands, and research on cultural artefacts. The questionnaire can also be requested at the journal’s discretion for any other submissions, even if these conditions are not met.  Please find more information on the policy and a link to download a blank copy of the questionnaire here: https://journals.plos.org/plosone/s/best-practices-in-research-reporting. Please upload a completed version of your questionnaire as Supporting Information when you resubmit your manuscript.

**Response:** Done and uploaded.

3. Thank you for stating the following financial disclosure:
“Région Bourgogne Franche-Comté (2020Y-22065 and 2022Y-13186)

National Council for Scientific and Technological Development–CNPq (200391/2022-4)”

Please state what role the funders took in the study. If the funders had no role, please state: "The funders had no role in study design, data collection and analysis, decision to publish, or preparation of the manuscript."
If this statement is not correct you must amend it as needed.
Please include this amended Role of Funder statement in your cover letter; we will change the online submission form on your behalf.

**Response:** The funder section is good and the funders had no rule for the study or manuscript. Please add "The funders had no role in study design, data collection and analysis, decision to publish, or preparation of the manuscript."

4. Thank you for stating the following in the Acknowledgments Section of your manuscript:
“The authors would like to thank the financial support from the Centre d’Expertise de la Performance from the Université de Bourgogne, the Région Bourgogne Franche-Comté (2020Y-22065 and 2022Y-13186), and the National Council for Scientific and Technological Development–CNPq (200391/2022-4). We thank all participants for their contribution and dedication to the study.”

We note that you have provided funding information that is not currently declared in your Funding Statement. However, funding information should not appear in the Acknowledgments section or other areas of your manuscript. We will only publish funding information present in the Funding Statement section of the online submission form.
Please remove any funding-related text from the manuscript and let us know how you would like to update your Funding Statement. Currently, your Funding Statement reads as follows:
“Région Bourgogne Franche-Comté (2020Y-22065 and 2022Y-13186)

National Council for Scientific and Technological Development–CNPq (200391/2022-4)”

Please include your amended statements within your cover letter; we will change the online submission form on your behalf.

**Response:** Authors have modified the acknowledgment section according to your comment.

5. In the online submission form, you indicated that “All the data generated and analysed as part of this study are not accessible to the public but are available on request.”

All PLOS journals now require all data underlying the findings described in their manuscript to be freely available to other researchers, either 1. In a public repository, 2. Within the manuscript itself, or 3. Uploaded as supplementary information.
This policy applies to all data except where public deposition would breach compliance with the protocol approved by your research ethics board. If your data cannot be made publicly available for ethical or legal reasons (e.g., public availability would compromise patient privacy), please explain your reasons on resubmission and your exemption request will be escalated for approval.

**Response:** Authors have modified the data availability statement. All data are available in a public repository (mendely.data) with the following DOI: 10.17632/3znpm68tyy.1

[Note: HTML markup is below. Please do not edit.]

Reviewers' comments:

**Response to Reviewer #1**

The English writing is good but there are a number of minor grammatical problems that should be fixed. I do not have time to identify all grammatical problems. If accepted hopefully the copy editors will help with that.

**Response:** Authors first thank the reviewer for the interesting and helpful comments. Please see below a point-by-point response to all of your comments. Alterations in the manuscript are presented in red font. Authors have carefully read and corrected the manuscript to limit grammatical problems.

Abstract
Line 41: “consisted of” not “consisted in”

**Response:** Corrected

Introduction
Line 69-70: The phrase “multiple neural alterations” is quite obtuse. Please be more specific.

**Response:** Some specific alterations have been given.

Line 79-80: Static stretching in general does not have detrimental effects on force production. It has been reported in a number of reviews that it is “prolonged” static stretching (typically more than 60-seconds per muscle group) that can induce performance impairments. (Behm and Chaouachi 2011, Kay and Blazevich 2012, Behm, Blazevich et al. 2016, Chaabene, Behm et al. 2019, Behm, Kay et al. 2021)

**Response:** Of course, authors agree with the reviewer comment. This sentence has been modified to make it clear for the reader.

Methods
Participants
Obviously, males and females are generally dichotomous in their anthropometric characteristics so providing the reader with one mean value will describe some hybrid male / female subject. You need to provide separate descriptions for each sex.

**Response:** Anthropometric description has been separated between women and men.

Line 123, 131, 201: Stand and reach test would provide an index of lower back and hamstrings flexibility but it would not be global. How does this test measure shoulder, neck flexibility for example? That type of test would be global. Please remove this term from the manuscript.

**Response:** The reviewer is right. We corrected this erroneous term. This term has also been removed in discussion.

Discussion
Line 345: Authors suggest this is a common result in the literature which is correct. Would it not be stronger to then cite reviews rather than two single original research papers. The review references provided at the end of this critique would be stronger evidence.

**Response:** As suggested by the reviewer, we changed references and only included review from your suggestions.

Line 351: It seems the only difference between your static stretching and neurodynamic stretching procedures was to flex and extend the neck while also performing dorsiflexion. I would agree that this action would have a greater emphasis on nerve stretching but the supine position with hip flexion and an extended knee would still provide tension on the musculotendinous structures. So I feel it is false to say it primarily affects nerve stiffness. It affects both!

**Response:** We agree with your comment and altered the part of the discussion.

Suggested references
Behm, D. G., A. J. Blazevich, A. D. Kay and M. McHugh (2016). "Acute effects of muscle stretching on physical performance, range of motion, and injury incidence in healthy active individuals: a systematic review." Appl Physiol Nutr Metab 41(1): 1-11.
Behm, D. G. and A. Chaouachi (2011). "A review of the acute effects of static and dynamic stretching on performance." Eur J Appl Physiol 111(11): 2633-2651.
Behm, D. G., A. D. Kay, G. S. Trajano and A. J. Blazevich (2021). "Mechanisms underlying performance impairments following prolonged static stretching without a comprehensive warm-up." Eur J Appl Physiol 121(1): 67-94.
Chaabene, H., D. G. Behm, Y. Negra and U. Granacher (2019). "Acute Effects of Static Stretching on Muscle Strength and Power: An Attempt to Clarify Previous Caveats." Front Physiol 10: 1468.
Kay, A. D. and A. J. Blazevich (2012). "Effect of acute static stretch on maximal muscle performance: a systematic review." Med Sci Sports Exerc 44(1): 154-164.

**Response to Reviewer #**

Thank you for the opportunity to review this manuscript. The authors examined the combined effects of passive warm-up and stretching. There is a lack of discussion on stretching. Also, sit and reach were used as a measure of flexibility, but comparing them to changes in the stiffness of the hamstrings is questionable.

**Response:** Authors thank the reviewer for the interesting and helpful comments. Please see below a point-by-point response to all of your comments. Alterations in the manuscript are presented in red font.

Abstract
Line 46-47: When was EMG measured? During the flexibility measurement?

**Response:** EMG was recorded during the maximal voluntary contractions. It has been added.

Line 47: What does “flexibility” indicate (maybe range of motion)? Authors should state specifically throughout the manuscript. Did the authors use the term "Range of motion" and "Flexibility" interchangeably?

**Response:** Throughout the manuscript we used these two different terms. They are not used interchangeably. Flexibility referred to some practical/functional tests such as the stand and reach used here. Range of motion referred to lab tests such as the passive knee extension used here. Authors have tried to better define terms and make it clear throughout the manuscript.

Line 53: If stiffness is measured, please describe it in the abstract method.

**Response:** Due to space restrictions, it was difficult to describe the stiffness assessment. However, we added it was evaluated during the passive knee extension test.

Introduction
Neurodynamic stretching is not a common type of stretching. Please explain in the introduction.

**Response:** As recommended, we added a sentence to indicate it is generally used in clinical settings.

Materials and Methods
Line 115: Why did you choose the effect size of 0.35? Please explain the rationale.

**Response:** Sorry it was a slight error. We did the a priori analysis in order to obtain a large effet (f = 0.40) with a power of 0.9. We corrected this mistake.

Line 120: Please explain the specific blinding procedure.

**Response:** We use and single-blind procedure. Both the experimenter and the volunteers knew the experimental conditions. The different data were collected, and files were coded. Blinded data were thereafter analyzed by one of the authors. Statistics were also conducted while being blinded. Coding was finally revealed after all statistics were done. No modification was done in the manuscript since authors think indicating data analyses and statistics were conducted blinded was sufficient enough.

Why was the stand-and-reach test used? This test includes the flexibility of the muscles other than the hamstrings (e.g., the trunk muscle). But, stretching was performed only on the hamstrings.

**Response:** Authors acknowledge, more muscle groups are considered. However, we decided to include this test to have a more "functional" result. By "functional", authors mean a test that could easily be applied in a real practice. This outcome is easily comprehensible by the practitioner. A sentence has been added in methods for clarity ('tests' part).

The room temperature of 45 degrees is high. Did the authors regulate water intake of participants?

**Response:** No regulation of water intake was made. Volunteers were free to drink as much water as they wanted. We added a sentence in the 'experimental design' paragraph.

Line 144: This study used six sets of 30 seconds of stretching. Previous studies have shown that more than 30 seconds lowers performance (Behm et al., 2011). Why did the authors use this stretching?

**Response:** Authors obviously know that prolonged static stretch could be detrimental for subsequent performance (e.g., force). We decided to apply such long stretch for the comparison with the neurodynamic nerve gliding procedure. In fact, this form of stretching scarcely studied is sports settings. We decided to have a rather long stretch to determine whether differences could be obtained between stretching procedures. The prolonged stretch duration has been acknowledged in discussion.

Results
Line 243: Please explain the reason for the discrepancy between the main effect for time and the post hoc analysis.

**Response:** The authors thank the reviewer for this comment. After a careful new check of our values, we noticed some small mistakes. First, we corrected two p-values in table 1. We forgot to take into account the sphericity correction for the ANOVA p-values. It was corrected for the maximal torque interaction (0.631 instead of 0.702) and for the time effect of the viscoelasticity (0.047 instead of 0.028). These corrections do not change our results. The other values have been double checked to avoid any other mistake. But the reviewer is right, a significant difference was obtained for post-hoc pairwise comparisons. No difference was obtained between PRE and WARM (P = 0.358) or between WARM and STRETCH (P = 0.681). However, a difference was obtained between PRE and STRETCH ((P = 0.025). The results section has been corrected accordingly

Discussion
Line 278-294 This study examined the combined effects of passive warm-up and stretching on range of motion and stiffness. The author describes the possibility that the changes in sit and reach may be related to tolerance, but I have a question about that because sit and reach includes not only the hamstring but also the trunk flexibility.

**Response:** The reviewer is right. As indicated previously, authors wanted a more functional / practical test that involved numerous muscle groups. This limitation is acknowledged at the end of the discussion.

Line 327 Reference 37 study did not use static stretching.

**Response:** Ref 37 investigated static stretching. However, the reviewer is right for ref 31. (ref 33 in the revised manuscript). Accordingly, authors deleted this reference from this sentence.

Previous studies have shown that static stretching for more than 3 minutes decreases stiffness (Matsuo et al., JSCR 2013; Takeuchi et al., JSSM, 2023). Muscle strength is also decreased by stretching for more than 30 seconds after warm-up (Behm 2011). Why did muscle strength not decrease despite a decrease in RMS? Of course, the results of this study are not necessarily the same as in previous studies, but the reasons why the results differ from many previous studies should be discussed in more detail.

**Response:** The lack of torque alteration was effectively uncommon in the literature. We tried to discuss such results by considering both sexes were included in the study. The large variability seen in fig 2A could be one likely explanation. We tried to include sex in the statistical analyses. The p-value of the 3-way ANOVA was close to 0.1 but remain unsignificant. We also did some new analyses with the sex effect for all variables. No main sex effect nor interactions were observed.

Please describe the limitations of this study.

**Response:** A limitation section has been added in 'discusion' section.
